# Biannual azithromycin distribution and child mortality among malnourished children: A subgroup analysis of the MORDOR cluster-randomized trial in Niger

**DOI:** 10.1371/journal.pmed.1003285

**Published:** 2020-09-15

**Authors:** Kieran S. O’Brien, Ahmed M. Arzika, Ramatou Maliki, Farouk Manzo, Alio K. Mamkara, Elodie Lebas, Catherine Cook, Robin L. Bailey, Sheila K. West, Catherine E. Oldenburg, Travis C. Porco, Benjamin Arnold, Jeremy D. Keenan, Thomas M. Lietman, for the MORDOR Study Group

**Affiliations:** 1 Francis I. Proctor Foundation, University of California, San Francisco, California, United States of America; 2 Division of Epidemiology, School of Public Health, University of California, Berkeley, California, United States of America; 3 The Carter Center, Niamey, Niger; 4 Clinical Research Unit, Department of Infectious and Tropical Diseases, London School of Hygiene & Tropical Medicine, London, United Kingdom; 5 Dana Center for Preventive Ophthalmology, Wilmer Eye Institute, Johns Hopkins University, Baltimore, Maryland, United States of America; 6 Department of Ophthalmology, University of California, San Francisco, California, United States of America; 7 Department of Epidemiology and Biostatistics, University of California, San Francisco, California, United States of America; 8 Institute for Global Health Sciences, University of California, San Francisco, California, United States of America; London School of Hygiene and Tropical Medicine, UNITED KINGDOM

## Abstract

**Background:**

Biannual azithromycin distribution has been shown to reduce child mortality as well as increase antimicrobial resistance. Targeting distributions to vulnerable subgroups such as malnourished children is one approach to reaching those at the highest risk of mortality while limiting selection for resistance. The objective of this analysis was to assess whether the effect of azithromycin on mortality differs by nutritional status.

**Methods and findings:**

A large simple trial randomized communities in Niger to receive biannual distributions of azithromycin or placebo to children 1–59 months old over a 2-year timeframe. In exploratory subgroup analyses, the effect of azithromycin distribution on child mortality was assessed for underweight subgroups using weight-for-age Z-score (WAZ) thresholds of −2 and −3. Modification of the effect of azithromycin on mortality by underweight status was examined on the additive and multiplicative scale. Between December 2014 and August 2017, 27,222 children 1–11 months of age from 593 communities had weight measured at their first study visit. Overall, the average age among included children was 4.7 months (interquartile range [IQR] 3–6), 49.5% were female, 23% had a WAZ < −2, and 10% had a WAZ < −3. This analysis included 523 deaths in communities assigned to azithromycin and 661 deaths in communities assigned to placebo. The mortality rate was lower in communities assigned to azithromycin than placebo overall, with larger reductions among children with lower WAZ: −12.6 deaths per 1,000 person-years (95% CI −18.5 to −6.9, *P* < 0.001) overall, −17.0 (95% CI −28.0 to −7.0, *P* = 0.001) among children with WAZ < −2, and −25.6 (95% CI −42.6 to −9.6, *P* = 0.003) among children with WAZ < −3. No statistically significant evidence of effect modification was demonstrated by WAZ subgroup on either the additive or multiplicative scale (WAZ < −2, additive: 95% CI −6.4 to 16.8, *P* = 0.34; WAZ < −2, multiplicative: 95% CI 0.8 to 1.4, *P* = 0.50, WAZ < −3, additive: 95% CI −2.2 to 31.1, *P* = 0.14; WAZ < −3, multiplicative: 95% CI 0.9 to 1.7, *P* = 0.26). The estimated number of deaths averted with azithromycin was 388 (95% CI 214 to 574) overall, 116 (95% CI 48 to 192) among children with WAZ < −2, and 76 (95% CI 27 to 127) among children with WAZ < −3. Limitations include the availability of a single weight measurement on only the youngest children and the lack of power to detect small effect sizes with this rare outcome. Despite the trial’s large size, formal tests for effect modification did not reach statistical significance at the 95% confidence level.

**Conclusions:**

Although mortality rates were higher in the underweight subgroups, this study was unable to demonstrate that nutritional status modified the effect of biannual azithromycin distribution on mortality. Even if the effect were greater among underweight children, a nontargeted intervention would result in the greatest absolute number of deaths averted.

**Trial registration:**

The MORDOR trial is registered at clinicaltrials.gov NCT02047981.

## Abstract 2

In this exploratory analysis of a randomized trial, Kieran O’Brien and colleagues investigate the effect of azithromycin in subgroups of malnourished children.

## Author summary

**Why was this study done?:**

- Previous research has found that distributing oral azithromycin to children 1–59 months old biannually reduces mortality in that age group.
- Despite the promise of this intervention to promote child survival in high-mortality settings, community distribution of antibiotics like azithromycin has the potential to increase antimicrobial resistance, which could reduce the efficacy of azithromycin and similar antibiotics over time.
- To harness the benefit of the intervention while reducing the potential for increasing resistance, some propose targeting the intervention to smaller groups of the population that face a particularly high risk of mortality.
- As malnutrition is implicated in a large proportion of childhood mortality, malnourished children are one potential subgroup to target.

**What did the researchers do and find?:**

- We conducted a subgroup analysis using data from a trial that compared communities randomized to azithromycin distribution or placebo distribution to children 1–59 months over 2 years in Niger.
- We included 27,222 children from the original trial who had weight measured at their first study visit and compared the effect of azithromycin to placebo on mortality among those who were not underweight, were underweight, and were severely underweight, and we calculated the number of deaths that would have been averted if this intervention had been given to the overall group as well as these groups defined by underweight status.
- The observed effect of azithromycin on mortality was strongest in the severely underweight subgroup, but we found no statistical evidence that the effect differed by underweight status.
- As only 10% of children were in the severe underweight group, the overall number of deaths averted would be greatest with an intervention treating all children 1–11 months, rather than only underweight children.

**What do these findings mean?:**

- The mortality rates and the observed effect of azithromycin on mortality were largest for the severe underweight group, suggesting a potential group to target with this intervention, although the study was not powered to detect this effect.
- Even if this study provided evidence that the effect was strongest among underweight children, a nontargeted intervention would have prevented the greatest number of deaths in this population.

## Introduction

Biannual azithromycin distribution reduced mortality among children 1–59 months of age in a large cluster-randomized trial in Malawi, Niger, and Tanzania (MORDOR trial, “Macrolides Oraux pour Réduire les Décès avec un Oeil sur la Résistance”) [1,2]. The strongest effects were observed in Niger, which had the highest baseline mortality rates, and in children 1–11 months of age [1]. In conjunction with existing child survival activities, this intervention has the potential to bolster progress in reducing under-5 mortality, particularly in high-mortality settings. However, these distributions increase the prevalence of antimicrobial resistance [3,4]. Limiting antibiotic distributions to smaller subgroups at the highest risk of mortality might be an approach to reduce selection for resistance [5].

Malnutrition is implicated in up to 45% of all childhood deaths globally [6]. Malnourished children are at increased risk of mortality from infectious diseases such as diarrhea and respiratory tract infections [6]. Moreover, the relationship between malnutrition and infection is complex, with undernutrition suppressing the immune system and increasing the risk of infection, and infection causing a reduction in appetite, malabsorption of nutrients, and competition for nutrients [7,8], Provision of antibiotics to malnourished children could lead to clearance of both overt and subclinical infections associated with mortality. Use of antibiotics with a long half-life, like azithromycin, could also prevent the development of infections during the 1–2 weeks after administration [9]. Other proposed mechanisms for a beneficial effect of antibiotics in undernourished children involve modulation of the intestinal microbiota, which could result in a reduction in gut flora that compete for nutrients and affect chronic conditions like environmental enteropathy [8,10–14].

Multiple studies have examined the role of antibiotics in malnourished children, with varying results. Three individual-randomized trials have compared antibiotics to placebo in the management of severe acute malnutrition [12,15,16]. One trial in Malawi found that children receiving antibiotics experienced greater nutritional recovery and less mortality than those receiving placebo [15], whereas two other trials found no difference in either nutritional recovery or mortality between arms [12,16]. Fewer studies have focused on children with moderate malnutrition, although one multi-country trial evaluating the effect of antibiotics on a number of outcomes in children with moderate acute malnutrition is currently underway [17].

Targeting high-risk subgroups such as malnourished children with azithromycin could preserve resources and lower the risk of selecting for antimicrobial resistance. However, evidence on the effect of antibiotics on mortality in malnourished children is mixed. The MORDOR trial provides an opportunity to examine the role of antibiotics in reducing mortality in malnourished children in a sub-Saharan African setting. The objective of this subgroup analysis was to assess whether the effect of biannual distribution of oral azithromycin on child mortality differed by nutritional status in Niger.

## Methods

### Trial design, setting, and participants

MORDOR was a large, simple, multisite cluster-randomized trial designed to compare the effect of biannual distribution of oral azithromycin to placebo on child mortality [1]. The protocol and statistical analysis plan for the main trial have been published, and the analyses presented here are exploratory [1]. This analysis included the Niger site, which enrolled communities in the Boboye and Loga districts (now Boboye, Loga, and Falmey districts after nation-wide redistricting). Communities with populations between 200 and 2,000 inhabitants according to the Niger 2012 census were eligible for inclusion in the main trial. Children 1–59 months of age who weighed ≥ 3.8 kg were eligible for treatment. This subgroup analysis included children 1–11 months old who had weight recorded at the time of the child’s first census, which could have been in any one of the censuses. Children 12–59 months old were excluded because crude height intervals were used to determine dose in children able to stand, and nutritional status indicators could not be accurately calculated for this group.

Ethical approval for the Niger site was obtained from the Niger Ministry of Health and the University of California, San Francisco Committee on Human Research. Verbal informed consent was obtained from households and caregivers before inclusion. The trial was conducted in accordance with the principles of the Declaration of Helsinki and was registered at Clinicaltrials.Gov (NCT02047981).

### Census

A door-to-door census was conducted every 6 months to enumerate households in the study area between December 2014 and August 2017. Demographic information (age, sex) was recorded for each child 1–59 months old. During follow-up census data collection, vital status (alive, dead, or unknown) and residence (living in community, moved outside community, or unknown) were recorded. Five censuses (four inter-census periods) were completed during the 2-year study. Data were collected electronically using a custom-designed mobile application (Conexus, Los Gatos, CA) and uploaded to a cloud-based server (Salesforce, San Francisco, CA).

### Interventions

At every biannual census, each child 1–59 months old was offered a single, directly observed dose of oral azithromycin or placebo (Pfizer, New York, NY). Children were given a dose of 20 mg per kg, which was assessed by height-stick approximation according to Niger’s trachoma program guidelines or by weight for children unable to stand. Children known to be allergic to macrolides were not treated. Adverse events were monitored and have been reported elsewhere [1,18].

### Outcomes

The outcome for this analysis is mortality, defined as community mortality rate (deaths per 1,000 person-years at risk). Data collected during the biannual census were used to assess the outcome. A death was included if a child was recorded as alive on one census and died at the subsequent census. Person-time at risk was calculated as the number of days between consecutive census periods or until death. Children who moved or had an unknown status at the subsequent census contributed half of the days during that inter-census period.

### Assessment of nutritional status

The trial protocol included assessment of weight for the purpose of determining dosage in children unable to stand. Trained study personnel recorded weight (if measured) and dose administered for all children in the mobile application. To determine dosage, children unable to stand were weighed (Amw-tl440 digital hanging scale, American Weigh Scales, Cumming, GA), and weight was recorded to the nearest 0.1 kg. A single weight measurement was taken at each visit. Age- and sex-adjusted weight-for-age Z-scores (WAZs) were calculated using the 2006 WHO Child Growth Standards with the zscorer package in R (R Foundation for Statistical Computing, Vienna, Austria) [19–21]. WAZ was dichotomized to group children without or with moderate to severe malnutrition (WAZ ≥ −2 and WAZ < −2, respectively) and without or with severe malnutrition (WAZ ≥ −3 and WAZ < −3, respectively). These categories were chosen to align with current classification standards used in nutritional policies and programs. Children with a baseline WAZ of less than −6 or greater than 5 were excluded according to WHO recommendations [20]. As WAZ was calculated after program completion, underweight children were not actively identified during the study period, and no additional measures were taken to address nutritional status during the trial.

### Randomization and masking

Within each country, communities were randomized 1:1 to receive biannual azithromycin or placebo. The randomization sequence was generated in R by the trial biostatistician and was implemented by unmasked members of the data team and Pfizer. The allocation was concealed by simultaneous randomization assignment. Participants, investigators, data collectors, and data analysts were masked to treatment assignment. Placebo was packaged to be identical in appearance to the azithromycin to maintain masking.

### Sample size and statistical methods

The MORDOR trial was designed and powered for the primary outcome, which has been previously published [1]. Briefly, the overall trial had 80% power to detect a 10% difference in all-cause mortality among communities receiving azithromycin compared to placebo, and the Niger site included 594 eligible communities [1]. Given the fixed design, the prevalence of moderate to severe and severe underweight, and the mortality rates within subgroups, this subgroup analysis had 80% power to detect additive interaction effects of the following sizes, interpreted as the mortality rate among underweight children receiving placebo in excess of the individual effects of underweight or placebo on mortality: 17 deaths per 1,000 person-years for the moderate to severe subgroup and 25 deaths per 1,000 person-years for the severe subgroup [22].

Analyses were conducted in R. Participant characteristics, WAZ, and outcomes were summarized by arm using frequency and percentage for categorical variables, mean and standard deviation for continuous variables, and incidence rate (deaths per 1,000 person-years, hereafter referred to as “mortality rate”) and 95% confidence interval for outcomes. Confidence intervals were constructed using percentiles from bootstrap resampling with 1,000 replicates. Participant characteristics were also compared among those included in the analysis and those excluded for having missing or invalid weight measurements. No multiple comparisons corrections were made.

Effect modification was evaluated non-parametrically with interaction contrasts [23]. To calculate the contrasts, subgroups were coded such that the groups with the lowest mortality rates were the reference categories (i.e., R_00_ = mortality rate among higher-weight children in communities assigned to azithromycin, R_01_ = mortality rate among underweight children in communities assigned to azithromycin, R_10_ = mortality rate among higher-weight children in communities assigned to placebo, and R_11_ = mortality rate among underweight children in communities assigned to placebo) [24]. An additive interaction contrast greater than 0 indicates the joint effect of receiving placebo and being underweight is greater than the sum of the individual effects considered separately. A multiplicative interaction contrast greater than 1 indicates the joint effect of receiving placebo and being underweight is greater than the product of the individual effects considered separately. The absolute number of deaths averted with azithromycin in each subgroup was also estimated using person-time at risk in both arms and the subgroup-level mortality rates.

Several sensitivity analyses were conducted. Survival probability was summarized by treatment arm and WAZ subgroup using Kaplan-Meier survival curves. Effect modification was also examined using Cox proportional hazards models. To determine the presence of multiplicative interaction, models included a shared frailty assuming a gamma distribution to account for clustering, the Efron method for ties, and treatment and WAZ as covariates with their product as an interaction term. Model estimates were reported with hazard ratios for each subgroup against a single reference category and with hazard ratios for the effect of treatment within each stratum of WAZ [23,25]. The estimated hazard ratios were used to calculate the Relative Excess Risk due to Interaction (RERI_HR_) to assess the presence and direction of additive interaction, with the same coding as used for the interaction contrasts [23–26]. The delta method was used to calculate standard errors for the RERI_HR_ [23]. As treatment arm was randomized and is the primary intervention of interest, confounding of the relationship between nutritional status and mortality was not considered, and no additional factors were controlled for in the models [23]. Model assumptions were evaluated graphically with ln(-ln) survival plots and analytically with tests of scaled Schoenfeld residuals as well as with models including terms for interactions with time to event for each covariate. The appropriateness of the distributional assumptions for the shared frailty were assessed by comparing results against models using a lognormal distribution for the shared frailty and estimated with generalized estimating equations (GEEs) to account for clustering.

Additional sensitivity analyses included evaluating the potential for bias introduced by the selection of the analysis sample by restricting the analysis to children eligible during the first inter-census period only and by restricting to children 1–5 months of age. To assess the impact of the use and form of WAZ, baseline weight, age, and sex were included in the models, and baseline WAZ was assessed in continuous form. To evaluate assumptions made in determining time to mortality when no exact date was available, an interval censoring method was also used. This was implemented as a generalized linear mixed model, with a binary outcome for death, a complementary log-log link, a term for inter-census period, and a random effect for community.

## Results

**Fig 1:**
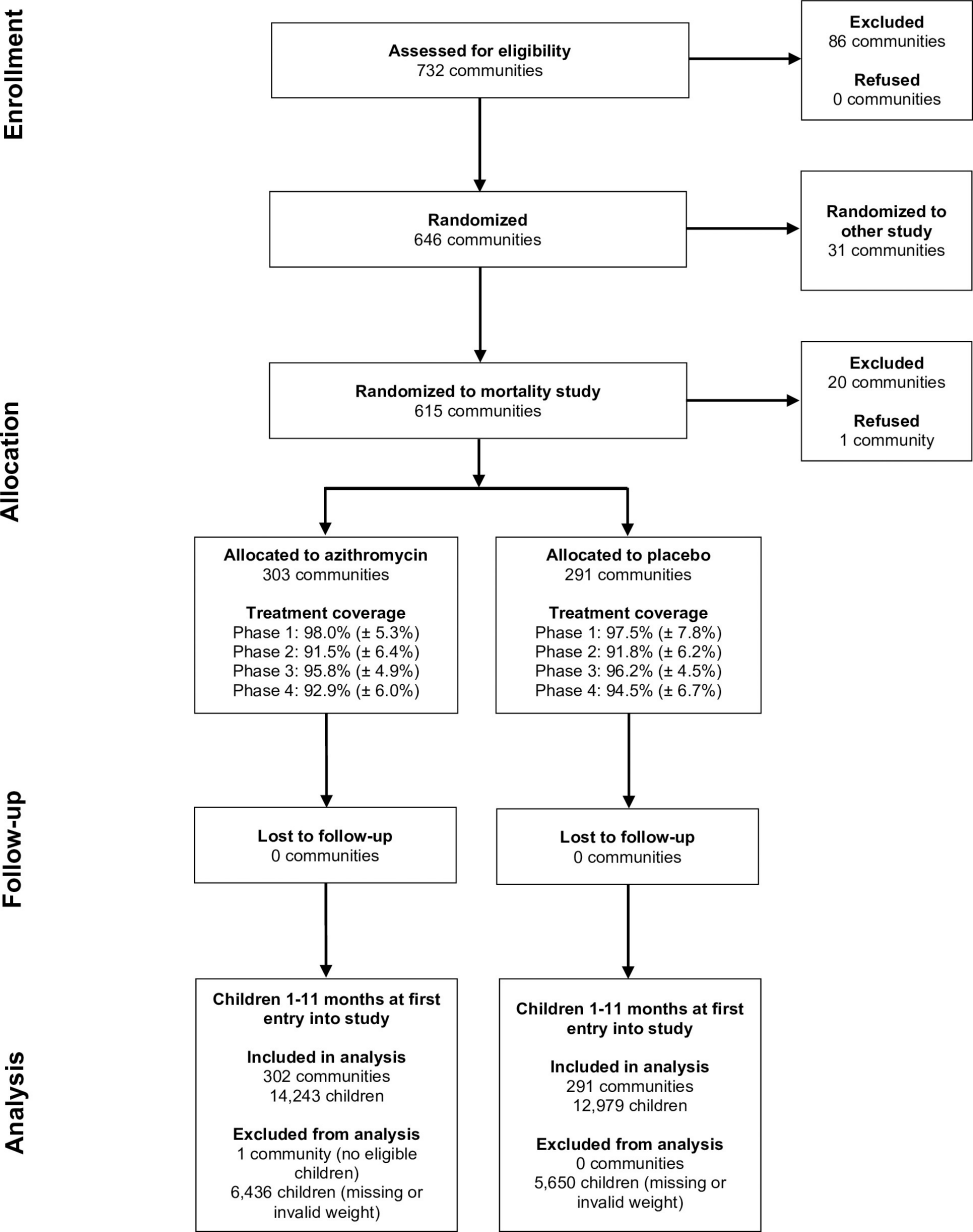
CONSORT participant flow diagram. CONSORT, Consolidated Standards of Reporting Trials.

In December 2014, 615 communities in Niger were randomized to receive biannual azithromycin or placebo in the main trial, of which 594 communities were successfully censused and included in analyses (Fig 1). Treatment coverage among children 1–59 months old was greater than 91% over the 4 inter-census periods in both arms. The final sample for this analysis included 593 communities with 27,222 children 1–11 months old who had a valid weight recorded at the time of the child’s first entry into the study. One community was not included because it had no eligible children, and 12,086 children 1–11 months old at their first census were excluded either for having no weight recorded (11,899 children, of which 10,271 had approximate height measured) or having a WAZ less than −6 or greater than 5 recorded (187 children). Over the 2-year study period, 5,189 children were lost to follow-up, with a similar percentage of children lost in each arm.

Characteristics of included children at the time of the child’s first census are shown by treatment arm in Table 1. Overall, the median age was 4 months (interquartile range [IQR] 3–6), and 49.5% of children (13,484/27,222) were female. Mean WAZ was −0.8 (SD 1.7), with 23.0% (6,268/27,222) of all children having a WAZ < −2 and 10.1% (2,755/27,222) having a WAZ < −3. All characteristics were similar in both arms. Excluded children were older than included children (median age 9 months, IQR 6–11), and a similar percentage were female (49.1%; S1 Table).

**Table 1:** Characteristics of children 1–11 months old with weight recorded at the time of entry into the study.

Characteristic	Azithromycin *n* = 14,243	Placebo *n* = 12,979
**Age, months, median (IQR)**	4 (3–7)	4 (3–6)
**Female sex, *n* (%)**	7,040 (49.4%)	6,444 (49.6%)
**Census period of entry into study, *n* (%)**		
1	4,470 (31.1%)	4,034 (31.4%)
2	3,880 (28.3%)	3,673 (27.2%)
3	2,751 (20.0%)	2,592 (19.3%)
4	3,142 (20.6%)	2,680 (22.1%)
**WAZ, mean (SD)**	−0.8 (1.7)	−0.8 (1.7)
**WAZ category, moderate to severe, *n* (%)**		
≥ −2	10,988 (77.1%)	9,966 (76.8%)
< −2	3,255 (22.9%)	3,013 (23.2%)
**WAZ category, severe, *n* (%)**		
≥ −3	12,796 (89.8%)	11,671 (89.9%)
< −3	1,447 (10.2%)	1,308 (10.1%)

**Abbreviations:** IQR, interquartile range; WAZ, weight-for-age Z-score

**Fig 2:**
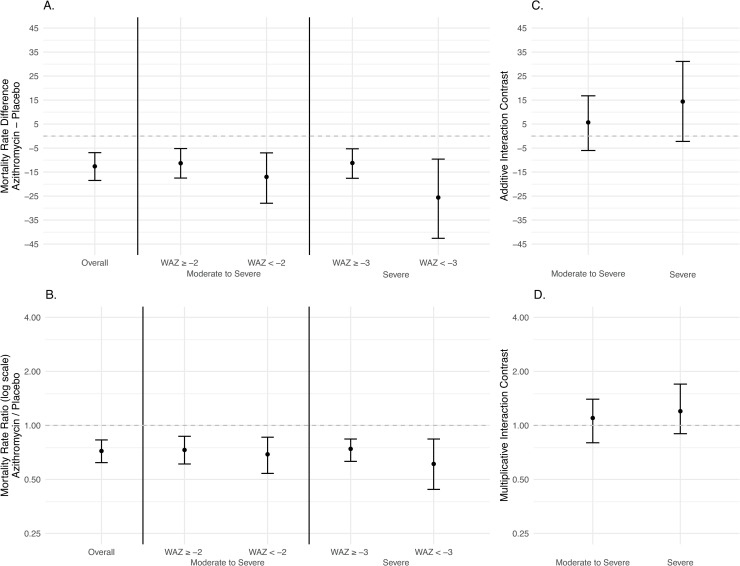
Comparison of mortality rates by treatment arm and WAZ subgroup with interaction contrasts. (A, B) Comparisons of mortality rate (deaths per 1,000 person-years) by treatment arm overall and by WAZ subgroup on the additive (A) and multiplicative (B) scales. (A) Mortality rate differences (mortality rate in communities assigned to azithromycin minus mortality rate in communities assigned to placebo). (B) Mortality rate ratios (mortality rate in communities assigned to azithromycin divided by mortality rate in communities assigned to placebo). (C, D) Interaction contrasts on the additive (C) and multiplicative (D) scales. Interaction contrasts defined subgroups such that the groups with the lowest mortality rates were the reference categories (i.e., R_00_ = mortality rate among higher-weight children in communities assigned to azithromycin, R_01_ = mortality rate among underweight in communities assigned to azithromycin, R_10_ = mortality rate among higher-weight children in communities assigned to placebo, and R_11_ = mortality rate among underweight children in communities assigned to placebo). (C) Interaction contrasts on the additive scale. (D) Interaction contrasts on the multiplicative scale. WAZ, weight-for-age Z-score.

The analysis included 1,184 deaths and a total of 30,852 person-years at risk (Table 2). The overall difference in the incidence of mortality comparing communities assigned to azithromycin to communities assigned to placebo was −12.6 deaths per 1,000 person-years (95% CI −18.5 to −6.9, *P* < 0.001). By subgroup, this difference was −17.0 (95% CI −28.0 to −7.0, *P* = 0.001) among those with WAZ < −2, and −25.6 (95% CI −42.6 to −9.6, *P* = 0.003) among those with WAZ < −3. Fig 2 compares mortality rates by treatment arm and subgroup. Interaction contrasts on the additive scale were 5.7 deaths per 1,000 person-years (95% CI −6.4 to 16.8, *P* = 0.34) for the moderate to severe subgroup and 14.4 deaths per 1,000 person-years (95% CI −2.2 to 31.1, *P* = 0.14) for the severe subgroup. On the multiplicative scale, these contrasts were 1.1 (95% CI 0.8 to 1.4, *P* = 0.50) and 1.2 (95% CI 0.9 to 1.7, *P* = 0.26), respectively. The estimated number of deaths averted with azithromycin among children 1–11 months old was 388 (95% CI 214 to 574) overall, 116 (95% CI 48 to 192) among children with WAZ < −2, and 76 (95% CI 27 to 127) among children with WAZ < −3.

**Table 2:** Number of deaths, person-time at risk, and mortality rates by treatment arm and subgroups of WAZ.

Category	Azithromycin				Placebo				Mortality rate ratio (95% CI)^2^	Mortality rate difference (95% CI)^2^
	*n*	Deaths	Person-years at risk	Mortality rate^1^ (95% CI)^2^	*n*	Deaths	Person-years at risk	Mortality rate^1^ (95% CI)^2^		
**Overall**	14,243	523	16,153	32.4 (29.3 to 35.5)	12,979	661	14,699	45.0 (40.3 to 49.7)	0.72 (0.62 to 0.83)	−12.6 (−18.5 to −6.9)
**WAZ category, moderate to severe**										
≥ −2	10,988	387	12,610	30.7 (27.0 to 34.4)	9,966	480	11,435	42.0 (36.9 to 47.3)	0.73 (0.61 to 0.87)	−11.3 (−17.5 to −5.2)
< −2	3,255	136	3,543	38.4 (32.4 to 44.9)	3,013	181	3,264	55.4 (46.7 to 64.9)	0.69 (0.54 to 0.86)	−17.0 (−28.0 to −7.0)
**WAZ category, severe**										
≥ −3	12,796	460	14,599	31.5 (28.3 to 35.0)	11,671	568	13,293	42.7 (37.6 to 47.6)	0.74 (0.63 to 0.84)	−11.2 (−17.6 to −5.3)
< −3	1,447	63	1,554	40.5 (30.9 to 49.7)	1,308	93	1,406	66.1 (53.7 to 79.4)	0.61 (0.44 to 0.84)	−25.6 (−42.6 to −9.6)

^1^Deaths per 1,000 person-years at risk.

^2^Confidence intervals calculated using bootstrap resampling at the community level to account for clustering; 1,000 replicates were used.

**Abbreviation:** WAZ, weight-for-age Z-score

**Fig 3:**
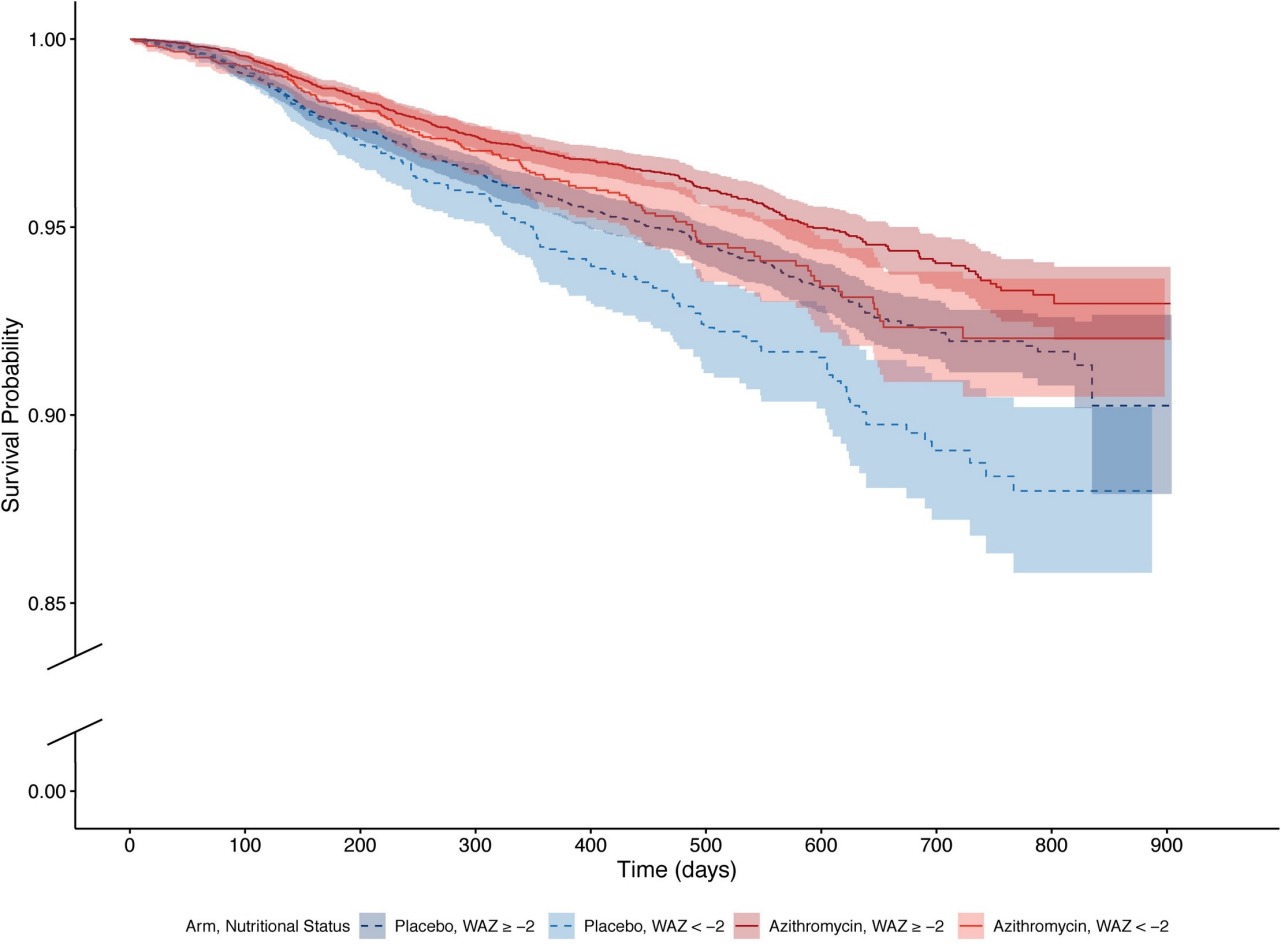
Kaplan-Meier estimates of survival probability by treatment arm and the moderate to severe underweight subgroup. Each curve depicts a different subgroup, with placebo represented by dotted lines in shades of blue and azithromycin represented by solid lines in shades of red. The darker shades indicate the higher-weight subgroup (WAZ ≥ −2), and the lighter shades indicate the underweight subgroup (WAZ < −2). The y-axis is broken for clarity and jumps from 0.00 to 0.85. WAZ, weight-for-age Z-score.

**Fig 4:**
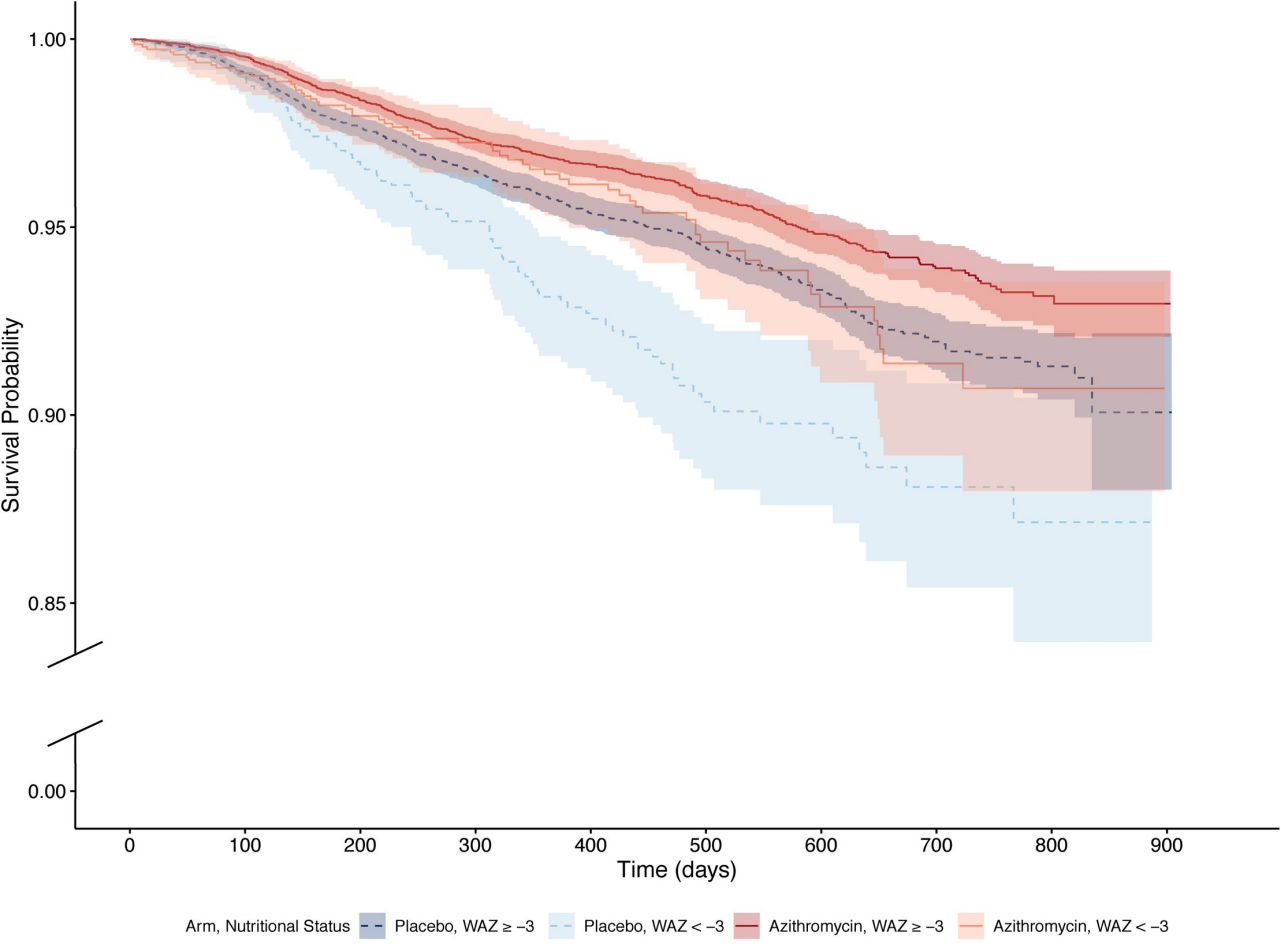
Kaplan-Meier estimates of survival probability by treatment arm and the severe underweight subgroup. Each curve depicts a different subgroup, with placebo represented by dotted lines in shades of blue and azithromycin represented by solid lines in shades of red. The darker shades indicate the higher-weight subgroup (WAZ ≥ −3), and the lighter shades indicate the underweight subgroup (WAZ < −3). The y-axis is broken for clarity and jumps from 0.00 to 0.85. WAZ, weight-for-age Z-score.

Figs 3 and 4 display survival probabilities by arm and subgroup, and Table 3 reports model-based estimates of mortality and effect modification by subgroup. Among children in placebo-treated communities, lower WAZ was associated with an increased hazard of mortality (HR 1.32, 95% CI 1.11–1.57, *P* = 0.002 comparing WAZ < −2 to WAZ ≥ −2 and HR 1.56, 95% CI 1.25–1.95, *P* < 0.001 comparing WAZ < −3 to WAZ ≥ −3). The hazard for mortality was lower in communities assigned to azithromycin than communities assigned to placebo, with a more pronounced effect for the subgroups of underweight children (27% lower in WAZ ≥ −2, 95% CI 15–38, *P* < 0.001; 30% lower in WAZ < −2, 95% CI 11–45, *P* = 0.003; and 38% lower in WAZ < −3, 95% CI 14–55, *P* = 0.005). When comparing underweight children in communities assigned to azithromycin to higher-weight children in communities assigned to placebo, the hazards for mortality were similar in both subgroups (HR 0.93, 95% CI 0.75–1.14, *P* = 0.48 comparing WAZ < −2 to WAZ ≥ −2 and HR 0.97, 95% CI 0.74–1.27, *P* = 0.82 comparing WAZ < −3 to WAZ ≥ −3). No evidence of effect modification was identified. Similar results were found in all sensitivity analyses.

**Table 3:** Sensitivity analysis using Cox proportional hazards regression to evaluate the association between biannual oral azithromycin distribution and mortality by WAZ subgroups^1^.

WAZ category	Hazard ratios (95% CI)			Measures of effect modification (95% CI)	
	Placebo	Azithromycin	Azithromycin within strata of WAZ	RERI_HR_ (additive)^2^	Ratio of HRs (multiplicative)
**Moderate to severe**					
≥ −2	1 (ref)	0.73 (0.62 to 0.85)	0.73 (0.62 to 0.85)	0.17 (−0.20 to 0.55)	0.96 (0.74 to 1.25)
< −2	1.32 (1.11 to 1.57)	0.93 (0.75 to 1.14)	0.70 (0.55 to 0.89)		
**Severe**					
≥ −3	1 (ref)	0.74 (0.64 to 0.85)	0.74 (0.64 to 0.85)	0.45 (−0.11 to 1.01)	0.84 (0.59 to 1.19)
< −3	1.56 (1.25 to 1.95)	0.97 (0.74 to 1.27)	0.62 (0.45 to 0.86)		

^1^As treatment arm was randomized and is the primary intervention of interest, confounding of the relationship between nutritional status and mortality was not considered, and no additional factors were controlled for in the model.

^2^For this calculation, subgroups were coded so the groups with the lowest mortality rates (azithromycin arm, higher WAZ subgroup) were the reference categories.

**Abbreviations:** HR, hazard ratio; RERI_HR,_ Relative Excess Risk due to Interaction from hazard ratios, WAZ, weight-for-age Z-score.

## Discussion

This subgroup analysis evaluated whether the effect of biannual azithromycin distribution on child mortality differed by underweight status in a high-mortality West African setting. Azithromycin was associated with an overall 28% reduction in mortality compared to placebo in children 1–11 months old with weight measured, similar to the age-based subgroup results from the main trial [1]. As expected given evidence on the relationship between malnutrition and mortality [6,27,28], lower weight for age was associated with increased mortality. The observed time to mortality in underweight children receiving azithromycin was approximately the same as that of higher-weight children receiving placebo. Although the absolute reduction in mortality between arms appears larger in both underweight groups, no evidence of effect modification by WAZ subgroup was found at the 95% confidence level. The number of deaths averted was greatest if all children were treated with azithromycin, regardless of nutritional status.

The nonspecific distribution of azithromycin to reduce child mortality presents an ethical dilemma: given the strong evidence of efficacy, it may be unethical to withhold such an intervention, yet the intervention’s effect on antimicrobial resistance warrants caution [29]. Increasing resistance could reduce the efficacy of essential antibiotics, potentially causing additional morbidity and mortality in the longer term. Targeting the intervention to high-risk subgroups is one solution to preserve resources and reduce negative consequences; targeting all children 1–11 months in this study population required 10 times the amount of azithromycin compared to targeting WAZ < −3. A targeted approach may also be more cost-effective than a broader distribution strategy [30]. The assumption that targeting vulnerable subgroups results in the greatest population health benefits has been questioned, however, since more lives are saved by intervening on a population with a wider risk spectrum [31–33]. Here, although there is some indication that intervening on those with the lowest WAZ may be particularly beneficial, the absolute number of deaths averted was 5 times greater when including all children 1–11 months as opposed to only the 10% with WAZ < −3. In addition, possible indirect effects might be lost with a more focused intervention. Finally, targeting a subgroup of the population presents its own ethical complexity, as providing a beneficial intervention more broadly might be more equitable when resources are available to do so [29].

Approximately 23% of the children included in this analysis were underweight, similar to other estimates indicating that Niger bears a high burden of malnutrition [34]. A single weight measurement was taken on a subset of children 1–11 months old who were unable to stand, which has several implications for interpretation of these results. First, other nutritional status indicators like wasting and stunting were not assessed, nor were the causes of underweight status. Underweight status has been shown to increase the risk of mortality in multiple settings [6,27,28,35,36], with some evidence demonstrating that WAZ alone is a highly sensitive and specific indicator of concurrent wasting and stunting [37]. In addition, as malnutrition is caused by a wide variety of factors, azithromycin might be more effective in cause-specific subgroups of underweight children, though this study was neither designed nor powered to assess the effect by smaller subgroups. Similarly, as underweight status could be a proxy for other child, household, and community characteristics, the mechanism of effect modification is likely more complex than modeled here. Second, although being underweight at the first visit likely predicts being underweight at later visits, we were unable to unable to examine the impact of changing nutritional status over time. Children who became underweight after their first visit thus might be misclassified by this analysis, which we would expect to bias any effect modification towards the null. Third, the selection of children 1–11 months of age who had weight measurements available could introduce bias, since children at the older end of that range who were able to stand were more likely not to be weighed. However, exclusions among the older age group were balanced by arm, overall and across census periods, and sensitivity analyses restricting the population to children 1–5 months produced similar results to the main analysis. Additionally, older children were not weighed. The analysis population thus might not be representative of the general population, as it might include a higher prevalence of underweight children and does not reflect the experience of children 12–59 months old. Fourth, the SD for WAZ was greater than 1 [20], likely due to measurement error since weight was assessed primarily for the purpose of intervention delivery. Only one measurement was taken at each visit for each child in order to determine dosage. As mean WAZ and SD were similar across arms, any information bias is likely to be conservative, which could have masked the presence of effect modification. Fifth, both mortality and malnutrition are known to vary seasonally in West Africa [38,39]. Seasonality-focused analyses were not pursued given the low power to further stratify the population, and the lack of an overall seasonal effect of azithromycin on mortality in the main trial [39]. Finally, the use of cutoffs to categorize malnourished groups has been criticized for creating a false separation of subgroups in which to intervene [40], particularly in high-burden areas where the entire distribution of anthropometric indicators is shifted downwards. As these cutoffs are actively used in current programs and policy, their use in this application provides readily available information to these sectors while also calling into question the impact of a targeted strategy that would exclude many children with mild to moderate malnutrition who also face an increased burden of mortality [27].

Additional limitations of this study include those shared by most subgroup analyses of trials, such as the potential for false negatives from lack of power and bias from use of improper subgroups. The effect sizes observed in this analysis were smaller than detectable by the design (5.7 versus 17 deaths per 1,000 person-years for the moderate to severe subgroup, and 14.4 versus 25 deaths per 1,000 person-years for the severe subgroup), indicating the analysis was underpowered. The use of baseline WAZ from children who entered the study after azithromycin had been distributed at the community level could result in bias since WAZ for these children is a post-randomization characteristic that could be influenced by treatment arm. A sensitivity analysis restricted to the first phase of the study did not reveal differences in results. Also, underweight prevalence did not differ by arm across census period, so more complex approaches to assessing or controlling for this potential bias were not pursued. In this type of dynamic cohort, differential loss to follow-up can result in selection bias. Although loss to follow-up was present, it was similar when compared by arm. Further research would be required to determine whether these results were generalizable to settings beyond those similar to Niger, which has a high burden of both malnutrition and mortality. Strengths of this study include the large sample size, the assessment of both additive and multiplicative interaction, and the randomized design.

In summary, a placebo-controlled trial found that biannual azithromycin distribution reduced mortality among children 1–11 months old regardless of underweight status. Although the observed mortality reduction with azithromycin was larger among subgroups of underweight children, underweight status was not a statistically significant effect modifier in this trial. Treatment of all children 1–11 months old would save 5 times as many lives as restricting treatments only to children with a WAZ < −3.

## Supporting information

S1 CONSORT Checklist Details about where the trial-specific information outlined by the CONSORT guidelines can be found in this manuscript. CONSORT, Consolidated Standards of Reporting Trials.
(DOC)

S1 Table Summary of characteristics of children 1–11 months old at the time of entry into the study among included children (*n* = 27,222) and excluded children (*n* = 12,086). Excluded children include those who were 1–11 months of age at the time of entry into the study and did not have weight measured (*n* = 11,899) or had an invalid weight recorded (*n* = 187).
(DOCX)

## Abbreviations

GEE: generalized estimating equation
IQR: interquartile range
MORDOR: Macrolides Oraux pour Réduire les Décès avec un Oeil sur la Résistance
RERI_HR_: Relative Excess Risk due to Interaction from hazard ratios
WAZ: weight-for-age Z-score
