# Supplementary material for: Biannual azithromycin distribution and child mortality among malnourished children: A subgroup analysis of the MORDOR cluster-randomized trial in Niger
Source: PLoS Med. 2020 Sep 15;17(9):e1003285. doi: 10.1371/journal.pmed.1003285 (PMC7491708; doi:10.1371/journal.pmed.1003285)
Supplement: S1 Table — Excluded children include those who were 1–11 months of age at the time of entry into the study and did not have weight measured (n = 11,899) or had an invalid weight recorded (n = 187). (DOCX) [file pmed.1003285.s002.docx]

**Supplementary Table 1.** Characteristics of children 1-11 months old at the time of entry among included versus excluded children.^1^

| **Characteristic** | **Included in analyses** | | | **Excluded from analyses^1^** | | |
| --- | --- | --- | --- | --- | --- | --- |
|  | **Azithromycin**  n = 14,243 | **Placebo**  n = 12,979 | **Total**  n = 27,222 | **Azithromycin**  n = 6,436 | **Placebo**  n = 5,650 | **Total**  n = 12,086 |
| Age, months , median (IQR) | 4 (3-7) | 4 (3-6) | 4 (3-6) | 9 (6-11) | 9 (6-11) | 9 (6-11) |
| Female sex , n (%) | 7,040 (49.4%) | 6,444 (49.6%) | 13,484 (49.5%) | 3,186 (49.5%) | 2,744 (48.6%) | 5,930 (49.1%) |
| Census period of entry into study, n (%) |  |  |  |  |  |  |
| 1 | 4,470 (31.1%) | 4,034 (31.4%) | 8,504 (31.2%) | 3,699 (57.5%) | 3,297 (58.4%) | 6,996 (57.9%) |
| 2 | 3,880 (28.3%) | 3,673 (27.2%) | 7,553 (27.7%) | 1,356 (21.1%) | 1,131 (20.0%) | 2,487 (20.6%) |
| 3 | 2,751 (20.0%) | 2,592 (19.3%) | 5,343 (19.6%) | 645 (10.0%) | 554 (9.8%) | 1,119 (9.9%) |
| 4 | 3,142 (20.6%) | 2,680 (22.1%) | 5,822 (21.3%) | 736 (11.4%) | 668 (11.8%) | 1,404 (11.6%) |

SD, standard deviation; WAZ, weight-for-age Z-score

**^1^** Excluded children are those who were 1-11 months old at the time of entry into the study (n = 12,086) and did not have weight measured (n = 11,899) or had an invalid weight recorded (n = 187).
